# Is semen a useful diagnostic tool for rare infections of the central nervous system?

**DOI:** 10.1186/1756-3305-5-297

**Published:** 2012-12-19

**Authors:** Ruqaiyyah Siddiqui, Naveed Ahmed Khan

**Affiliations:** 1 Department of Biological and Biomedical Sciences, Aga Khan University, Stadium Road, Karachi, Pakistan

## Abstract

Given that the blood-testis barrier is more permeable than the blood–brain barrier, the use of semen to detect rare parasitic antigens/infections of the CNS in males is hypothesized.

## 

Infections of the central nervous system (CNS) can progress rapidly and cause substantial damage or even death if they are not diagnosed promptly and treated aggressively. Given the involvement of the CNS, it is typical to use cerebrospinal fluid for the identification of infectious agents/antigens, and the host factors to suspected disease. In general, pathogens colonize mucosal surfaces, followed by invasion of the intravascular space leading to haematogenous spread and entry into the CNS at the sites of the blood–brain barrier (BBB) or blood-cerebrospinal fluid (CSF) barrier at the choroid plexus. The use of CSF specimens has been of tremendous value in the diagnosis of CNS infections but the rarity of pathogens/antigens in the CSF in some of the parasitic infections such as neurocysticercosis, cerebral toxoplasmosis, granulomatous amoebic encephalitis due to *Acanthamoeba* and *Balamuthia mandrillaris* and primary amoebic meningoencephalitis due to *Naegleria fowleri* has made accurate diagnosis challenging [1,2]. For such infections, the CSF specimen is generally condensed by centrifugation, followed by microscopic observation for parasites or immunodiagnosis. While there are many similarities between the endothelial cells of the testis and the brain, several lines of evidence suggest that the blood-testis barrier (BTB) exhibits a higher degree of permeability compared with the BBB [3-5]. Physiological experiments have shown that tracers like L-glucose, sucrose, and albumin that do not ordinarily cross the BBB, are readily detected in the lymphatics draining the testis [3]. The BBB is tightly regulated by the presence of tight junctions between endothelial cells of microvessels, while the BTB is restrictive due to the adjacent Sertoli cells near the basement membrane of the seminiferous tubules. Based on these findings, it is plausible that parasites/antigens cross the semipermeable testicular blood vessels readily, in addition to the highly selective BBB. Given the condensed nature of semen specimens (unlike urine which may be useful to diagnose overwhelming bacterial infections), here we hypothesize the use of semen to detect rare parasitic infections of the CNS, perhaps at a higher density than the CSF. If the results correlate with the CSF findings and the neuroimaging data, it could provide a useful tool in the diagnosis of rare parasitic CNS infections in males. Although our theory to use semen to detect rare parasitic infections of the CNS has a scientific basis, intensive future research is needed to test this theoretical hypothesis before it is widely accepted by the scientific community.

## Competing interests

The authors declare that they have no competing interests.

## Authors' contributions

Both authors contributed equally to this manuscript and both read and approved its final version.
